# Assessing the Usefulness of Mobile Apps for Noise Management in Occupational Health and Safety: Quantitative Measurement and Expert Elicitation Study

**DOI:** 10.2196/46846

**Published:** 2023-11-14

**Authors:** Jingchen Huyan, Chandnee Ramkissoon, Mah Laka, Sharyn Gaskin

**Affiliations:** 1 Adelaide Exposure Science and Health, School of Public Health, The University of Adelaide, Adelaide, Australia; 2 Adelaide Health Technology Assessment, School of Public Health, The University of Adelaide, Adelaide, Australia

**Keywords:** occupational health, noise management, mobile applications, mobile apps, mHealth, Mobile App Rating Scale, MARS, management, hearing, hearing loss, mobile phone, noise detection, usefulness, tool

## Abstract

**Background:**

Overexposure to occupational noise can lead to hearing loss. Occupational noise mapping is conventionally performed with a calibrated sound level meter (SLM). With the rise of mobile apps, there is a growing number of SLM apps available on mobile phones. However, few studies have evaluated such apps for accuracy and usefulness to guide those with occupational noise detection needs in selecting a quality app.

**Objective:**

The purpose of this study was to evaluate the accuracy and usefulness of SLM mobile apps to guide workplace health and safety professionals in determining these apps’ suitability for assessing occupational noise exposure.

**Methods:**

The following three iOS apps were assessed: the NIOSH (National Institute for Occupational Safety and Health) Sound Level Meter, Decibel X, and SoundMeter X apps. The selected apps were evaluated for their accuracy in measuring sound levels in low-, moderate-, and high-noise settings within both simulated environments and real-world environments by comparing them to a conventional SLM. The usefulness of the apps was then assessed by occupational health specialists using the Mobile App Rating Scale (MARS).

**Results:**

The NIOSH Sound Level Meter app accurately measured noise across a range of sound levels in both simulated settings and real-world settings. However, considerable variation was observed between readings. In comparison, the Decibel X and SoundMeter X apps showed more consistent readings but consistently underestimated noise levels, suggesting that they may pose a risk for workers. Nevertheless, none of the differences in sound measurements between the three apps and the conventional SLM were statistically significant (NIOSH Sound Level Meter: *P*=.78; Decibel X: *P*=.38; SoundMeter X: *P*=.40). The MARS scores for the three apps were all above 3.0, indicating the usefulness of these apps.

**Conclusions:**

Under the conditions of this study, the NIOSH Sound Level Meter app had equivalent accuracy to the calibrated SLM and a degree of usefulness according to the MARS. This suggests that the NIOSH Sound Level Meter app may be suitable for mapping noise levels as part of a monitoring strategy in workplaces. However, it is important to understand its limitations. Mobile apps should complement but not replace conventional SLMs when trying to assess occupational noise exposure risk. Our outcomes also suggest that the MARS tool may have limited applicability to measurement-based apps and may be more suited to information-based apps that collect, record, and store information.

## Introduction

More than 1.5 billion people worldwide live with varying degrees of hearing loss, with nearly 500 million living with severe hearing loss [1]. Nearly 16% of these adults have severe hearing loss as a result of occupational noise exposure [2]. In Australia, over 111,000 people have occupational noise-induced hearing loss (ONIHL) [3], resulting in a loss of 62,218 quality-adjusted life years and 135,561 productivity-adjusted life years. The projected welfare-based loss is Aus $5.5 billion (US $3.5 billion), and the projected productivity-based loss is Aus $21.3 billion (US $13.4 billion) [3].

ONIHL is preventable, and the economic benefits of intervention are considerable. According to the modelling study on the productivity burden of ONIHL in Australia by Si et al [3], even a modest reduction of overall noise exposure in the workplace can significantly reduce the socioeconomic burden of ONIHL. The prevention of ONIHL can be achieved through occupational noise awareness and exposure control and monitoring [4].

The Australian national standard for occupational noise levels is an average daily exposure to ≤85 A-weighted dB (dB[A]; ie, an 8-h, A-weighted equivalent continuous sound level [LAeq] of ≤85 dB) [5]. Noise assessment in the workplace is typically done through the use of a handheld sound level meter (SLM); the SLM is placed 10 to 20 cm from the worker’s ear canal for a representative period of time, during which routine tasks are undertaken. Owning a conventional SLM may be prohibitively expensive for most small- and medium-sized enterprises, resulting in difficulties with taking timely noise management measurements and evaluating interventions. With the increasing number of smartphones worldwide, there is potential for increased accessibility to noise mapping via mobile apps with noise monitoring capabilities. Often, mobile apps with SLM features are used to complement traditional SLMs. However, the accuracy of such apps in assessing and monitoring occupational noise exposure is not well evaluated.

The evidence for the accuracy of SLM mobile apps appears limited to simulated laboratory studies with limited evidence of accuracy based on real-world scenarios. The results of one simulation study showed that mobile SLM apps accurately measured 65 dB to 95 dB of pink noise (defined as random noise having equal energy per octave), with an error of approximately 2 dB(A) [6]. Another study showed the accuracy of mobile SLM apps in measuring white and pink noises from 3 sound sources, namely conversation, occupational steelmaking, and conveyor belt operation [7]. A study by Murphy and King [8] compared the accuracy of 7 SLM apps on 100 smartphones (both Android and iOS) in detecting white noise (containing many frequencies). The study showed a difference in noise level assessment between the two types of phones; Apple phones showed a measurement error within 1 dB(A), while Android phones showed twice the variation in noise measurement. Although these studies evaluated the accuracy of mobile apps from different perspectives, the sound sources in these studies were simulated in a laboratory. The accuracy of such apps in a real-world noise scenario, which represents a more realistic noise exposure scenario for workers, has yet to be demonstrated. In this study, the accuracy of mobile apps for assessing occupational noise exposure in simulated and real-world settings was evaluated according to the Occupational Health Hazard Management framework [9].

In addition to accuracy, user experience and usefulness are also key considerations for SLM mobile apps, as they may influence the readiness of users to choose one particular noise measurement app over others. Several guidelines and scales are available for assessing the effectiveness of digital technologies in health care settings, such as the Xcertia Guidelines from the American Medical Association, the Digital Technology Assessment Criteria from National Health Service England, and the Mobile App Rating Scale (MARS) [10,11]. A growing number of studies use the MARS to assess the usefulness of health-related mobile apps, such as food allergy management apps, blood disease management apps, diabetes management apps, and occupational therapy apps [12-15]. However, to our knowledge, the MARS has not previously been applied to assess occupational health-related mobile apps.

This study aimed to evaluate the accuracy and usefulness of SLM mobile apps for an occupational health context. The objectives of this study were (1) to assess the accuracy of 3 SLM apps by comparing them with conventional SLM instrumentation in simulated noise situations within a laboratory and in a real-world noise environment and (2) to assess the usefulness of the studied apps by using the MARS. The study results could help influence the adoption of high-quality SLM apps in occupational settings through empirical evidence and informed decision-making by relevant health specialists.

## Methods

### Accuracy of SLM Mobile Apps

The accuracy of mobile SLM apps in measuring noise levels, when compared to a conventional SLM, was assessed in the following two contexts: laboratory and real-world conditions.

#### Selection of SLM Mobile Apps

**Figure 1.**
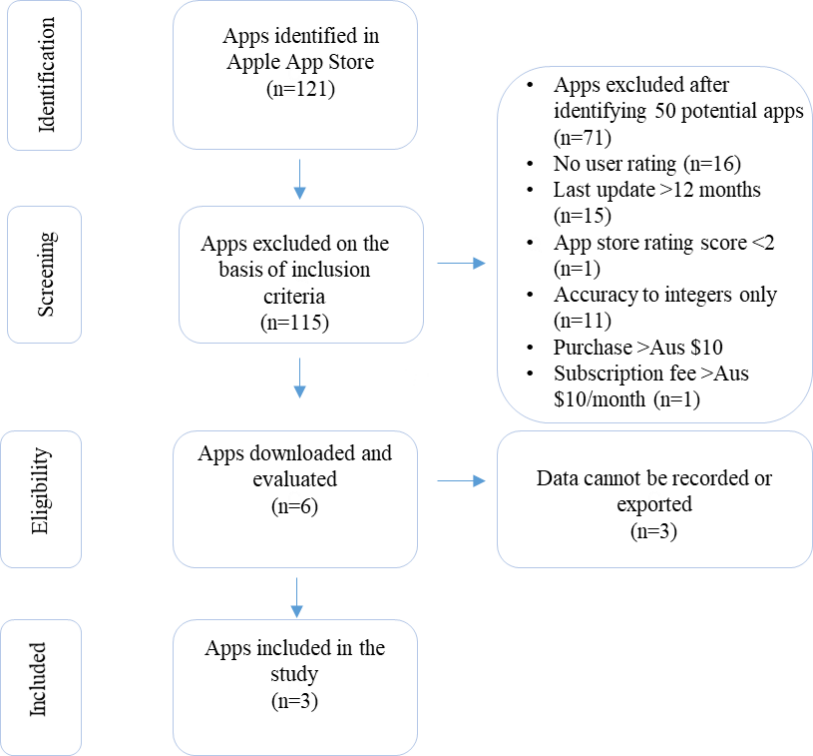
Flow diagram of the selection process for the apps included in this study. A currency exchange rate of Aus $1=US $0.63 is applicable.

An Apple device (iPhone 13 Pro, iOS 15.6; Apple Inc) was used in this study because the built-in hardware (microphone, circuitry, and signal processing hardware) and operating system showed less variability across models when compared to Android phones [16]. SLM apps were systematically searched for and screened according to the inclusion and exclusion criteria in Figure 1. The specific app inclusion criteria were (1) app store review scores of >1, (2) the app was last updated within 12 months, (3) an app store score of >2, (4) a reading precision of >1 decimal place, (5) the app costs less than Aus $10 (US $6.31) or the subscription costs less than Aus $10 (US $6.31) per month, and (6) the app has the ability to log and export data externally. The app store score and review scores were selected as proxies for the accuracy of apps to ensure that apps regarded as useful or accurate by most users were included in our analysis. We understood that the app review score may not be a consistent criterion for measuring app quality; therefore, we used a review score of >1 and an app store score of >2 as the initial gatekeeper criteria to select apps that were used and regarded as fairly accurate by many users. This scoring system did not impact the app accuracy assessment of the experts but was used as an app selection criterion. The following three apps met all criteria and were included in this study: Decibel X, NIOSH (National Institute for Occupational Safety and Health) Sound Level Meter, and SoundMeter X. Details about the specifications of the three apps are provided in Multimedia Appendix 1.

#### Laboratory Simulation

This study was carried out in a room at a university where the background noise level was 30 dB(A). A loudspeaker was used to reproduce a standard white noise generated from a web-based tone generator [17]. The sound level of the white noise was increased in increments from 60 dB to 85 dB, using an amplifier (Marshall Emberton portable speaker; Zound Industries).

The sound levels were measured by using a conventional SLM (Type 2250; Brüel & Kjær) and the mobile phone, which were placed side by side and approximately 100 cm away from the noise source. Both devices were activated and stopped simultaneously. The experiment was replicated 3 times, using 1 app each time. Measurements were taken for 30 seconds, and an average LAeq, in dB, was logged.

#### Real-World Simulation

The sound mapping for the real-world study was conducted across 3 different sites in a tertiary education environment, namely a library, a busy student and staff activity center, and an engineering workshop facility where a high-pressure water-jet cutting machine (Techni Waterjet i35-G2; Techni Ltd) was in use. These three locations represented a range of noise levels—low sound levels at the library (45-50 dB; typically quiet radio music and normal conversation), moderate sound levels at the student and staff activity center (60-70 dB; typically loud conversation), and high sound levels at the engineering workshop facility (75-85 dB; typically heavy traffic or a front-end loader) [5]. Similar equipment and methods to the laboratory simulation study were used; briefly, sound levels in each environment were measured for 30 seconds (LAeqs were logged) with a mobile app on the same Apple device (iPhone 13 Pro, iOS 15.6) alongside the conventional SLM. A total of 20 samples were collected from each location and each app. After the two simulation studies, the accuracy of the mobile apps was evaluated by comparing the LAeqs taken from the mobile apps to those taken from the conventional SLM.

### Usefulness of the Mobile Apps

The usefulness of the SLM apps was evaluated by 3 occupational health specialists using the MARS; the specialists rated the apps based on the five components of the MARS—engagement, functionality, aesthetics, information, and subjective quality. The MARS was selected for this study because it is reported as one of the most widely used tools for evaluating the quality of mobile apps, and validation studies have demonstrated its suitability for quality assessment [18]. The five components of the scale were further divided into 22 items, as follows: engagement was divided into entertainment, interest, customization, interactivity, and target group; functionality was divided into performance, ease of use, navigation, and gestural design; aesthetic was divided into layout, graphics, and visual appeal; information was divided into accuracy, goals, quality and quantity of information, visual information, and credibility; and subjective quality was divided into recommendation, frequency of use, willingness to pay, and overall rating. Each MARS item was scored by using a 5-point Likert scale (1=inadequate; 2=poor; 3=acceptable; 4=good; 5=excellent). The MARS questionnaire was reworded to adapt it to SLM apps.

### Ethical Considerations

The three health specialists were chosen from a professional membership database—the Australian Institute of Occupational Hygienists—based on their expertise in occupational noise exposure assessment. The selected health specialists were invited to participate in this study via publicly available information, such as email addresses. Informed consent was obtained from all participants involved in this study. The consent form provided details such as the objectives of this study, information on the team of investigators, the kind of participation expected, and the nature of survey. Only when potential participants gave consent to participate in this study, they were emailed the survey link. They were further deidentified to maintain the anonymity of the responses. This study was approved by the University of Adelaide Human Research Ethics Committee (approval number: H-2022-196). Participants did not receive any compensation for their participation.

### Statistical Analysis

This study used Stata software (version 17; StataCorp LLC) for data analysis, and both parts of the project were analyzed separately. The difference in measurements of sound levels between the SLM apps and the conventional SLM and the variability within the apps were assessed for statistical significance in the laboratory and real-world studies via a rank Mann-Whitney *U* test. The samples were first tested for normality and equal variances via a Shapiro-Wilk test (sample size of <50). Given that the data collection method involved using only 1 app and 1 SLM at the same time rather than using all apps and the SLM at the same time, differences between mobile apps were not considered in this study. For the second part of this study, the scores obtained for each component of the MARS quality assessment were averaged among the three evaluators.

## Results

### Accuracy of SLM Mobile Apps

#### Laboratory Simulation

**Figure 2.**
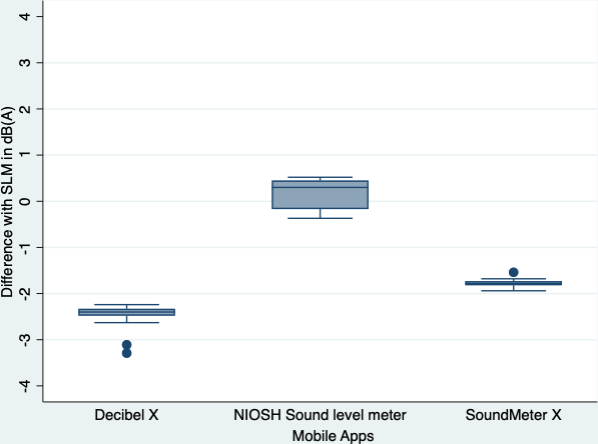
Distribution of the differences between the measured values of the three apps—Decibel X, NIOSH SLM, and SoundMeter X—and those of the conventional SLM in the laboratory simulation study. dB(A): A-weighted dB; NIOSH: National Institute for Occupational Safety and Health; SLM: sound level meter.

All 3 apps gave different readings when compared to the conventional SLM but to various extents (Figure 2). The readings of the NIOSH Sound Level Meter app were the most accurate, with measurement readings within 0.5 dB(A) of the conventional SLM readings (Figure 2). However, more variation in the NIOSH Sound Level Meter measurements was observed when compared to the other two apps tested (around 2 dB[A]). In contrast, the Decibel X app data showed the least variability when compared to the other two apps but recorded lower noise levels than those recorded by the conventional SLM (>2 dB[A] deviation). SoundMeter X also consistently recorded lower sound levels than those recorded by the conventional SLM (Figure 2). However, none of the differences observed between the apps and the conventional SLM were statistically significant (NIOSH Sound Level Meter: *P*=.78; Decibel X: *P*=.38; SoundMeter X: *P*=.40).

#### Real-World Simulation

**Figure 3.**
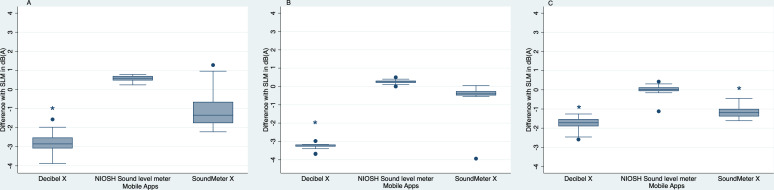
Distribution of the differences between the measured values of the three apps and the conventional SLM in (**A**) low-noise (40-50 dB), (**B**) moderate-noise (55-70 dB), and (**C**) high-noise (75-85 dB) settings in the real-world study. dB(A): A-weighted dB; NIOSH: National Institute for Occupational Safety and Health; SLM: sound level meter. *Statistical difference between the app and the conventional SLM at a 95% CI.

The results in the real-world study showed similar trends to the laboratory study. The NIOSH Sound Level Meter app results differed minimally from the conventional SLM results across the different sound level ranges; hence, it was considered to be the most accurate app overall (Figure 3). SoundMeter X was observed to be more accurate than Decibel X in all 3 locations (Figure 3).

In the low-noise (40-50 dB) location, the SoundMeter X and Decibel X apps underestimated sound levels by approximately 2 dB(A) and 4 dB(A), respectively, with Decibel X readings being statistically different (*P*<.001) from the readings of the conventional SLM (Figure 3A). In the moderate-noise setting (55-70 dB), the NIOSH Sound Level Meter app results differed minimally, by approximately 0.5 dB(A), from those of the conventional SLM, and the app’s data were more reliable than those of the other two apps (Figure 3B). One measurement of SoundMeter X was an outlier, with a >4-dB(A) underestimation when compared to the conventional SLM, which was attributed to a brief app malfunction; all other measurements of the SoundMeter X app differed minimally, by approximately 0.5 dB(A), from the results of the conventional SLM (Figure 3B). In contrast, the Decibel X measurements differed significantly (*P*<.001) from the conventional SLM measurements, underestimating sound level by >3 dB(A) (Figure 3B).

In the high-noise setting (the engineering workshop), the NIOSH Sound Level Meter app results were again the most accurate and consistent, differing minimally (by 0.5 dB[A]) from the conventional SLM results (Figure 3C). The SoundMeter X app recorded 2 significant outliers (excluded from this data set), suggesting unreliable app functioning. In addition, the Decibel X app data differed (by >3 dB[A]) from the conventional SLM data. Both SoundMeter X and Decibel X significantly underestimated the noise levels when compared to the conventional SLM (*P*<.001) (Figure 3C).

### Usefulness of the Mobile Apps

The MARS survey results were summarized as an average of the experts’ ratings, and these are shown in Table 1. Further information about the scores is available in Multimedia Appendix 2. Overall, Decibel X had the highest average MARS score (4.0) among all 3 apps. Table 1 shows that the NIOSH Sound Level Meter app had the highest scores in the functionality and information sections—4.3 and 4.2, respectively—which assess the functionality of an app and the reliability of information, respectively. However, the overall MARS score for this app (3.5) was the lowest among the three apps rated by the experts. Interestingly, Decibel X, which was the least accurate app in the accuracy assessment, had the highest overall score when compared to the other two apps, with higher scores in the engagement and subjective quality domains—4.4 and 4.0, respectively (Table 1). According to the 5-point Likert scale, apps with an overall score of >3.0 are considered acceptable in terms of usefulness. Since the overall scores were all higher than 3.0, the three apps could be considered useful according to this scale.

**Table 1.** Summary of the average MARS^a^ scores for the three mobile apps.

Mobile app	Usefulness domain score, mean					
	Engagement	Functionality	Aesthetics	Information	Subjective quality	Overall score
NIOSH^b^ Sound Level Meter	3.2	4.3	3.2	4.2	3.3	3.5
Decibel X	4.4	3.9	4.2	3.6	4.0	4.0
SoundMeter X	3.7	3.8	3.6	3.7	3.2	3.6

a MARS: Mobile App Rating Scale.

b NIOSH: National Institute for Occupational Safety and Health.

## Discussion

### Principal Results

This study evaluated the accuracy and usefulness (user experience) of SLM mobile apps to guide workplace health and safety professionals in determining these apps’ suitability for assessing occupational noise exposure. It is the first reported application of the MARS to the assessment of occupational health-related mobile apps. Furthermore, this study builds upon previously published accuracy studies by examining app performance in realistic working environments, not just in a laboratory simulation.

In terms of the overall performance of the apps tested across all elements of this study, the NIOSH Sound Level Meter app was consistently the most accurate mobile app in measuring noise levels ranging from 40 dB(A) to 85 dB(A). In comparison, Decibel X was the least accurate in similar noise settings, suggesting that it is not a reliable tool for measuring noise in occupational settings. The Decibel X app had the highest overall MARS score, but the individual domain scores indicated that although this app might have superior aesthetics, graphic layouts, interactivity, and visual appeal, users considered it inferior to other apps in terms of the quality and credibility of information. This aligns with our finding that Decibel X was the least accurate app in all 3 occupational settings when compared against the conventional SLM. The SoundMeter X app gave more accurate measurements than those provided by Decibel X but showed a high level of variability in the real-world environment, particularly in low-noise (40-50 dB[A]) settings, suggesting that it may not be sensitive and reliable enough for effective noise exposure assessments. Therefore, under the conditions of this study, the NIOSH Sound Level Meter app had comparable accuracy to a conventional SLM and could be used to acquire and monitor real-time noise exposure data, which could be used to raise occupational awareness about the potential hazards to hearing in a work environment. The experts in our study considered the NIOSH Sound Level Meter app useful; however, its overall aesthetics, including visual appeal, graphics, and layout, were scored the lowest among all 3 apps. This indicated that the NIOSH Sound Level Meter app could be improved at the aesthetics and user engagement levels.

According to IEC (International Electrotechnical Commission) standard 61672, class 2, a noise measurement device should have an error within 2 dB(A) [19]. The results from this study demonstrated that the NIOSH Sound Level Meter and SoundMeter X apps complied with this international standard. There were, however, differences noted in performance between simulation outcomes and real-world outcomes. The NIOSH Sound Level Meter app had greater variability in noise measurements under real-world conditions than under laboratory simulation conditions. In contrast, the other two apps showed the opposite effect—more reproducible data in the real world than in the laboratory—but both consistently underestimated noise levels. In the context of occupational noise assessment, it would be more preferable to overestimate sound levels than to underestimate them when informing protective effects for the health of the target population. Moreover, despite the overall MARS score of the NIOSH Sound Level Meter app being the lowest among the three apps tested, the app did obtain an overall score of >3.0 and the highest functionality and information scores. Thus, the NIOSH Sound Level Meter app had a degree of usefulness, especially for providing reliable information and appropriate functionality in our study settings.

The advantages that a mobile app might have over a conventional SLM include cost (often free), ease of access, and simple operation. The NIOSH Sound Level Meter app may be suitable for scenarios where a rapid assessment is required (eg, a change in task or setting up new equipment), while a conventional SLM may be important for scenarios where precise analysis is required, such as frequency analysis or worker exposure risk assessment. Our results suggest that SLM apps can complement traditional SLMs in occupational noise detection but may not be a substitute for conventional SLMs.

The accuracy results of the apps assessed in this study appear consistent with those of previous studies. The NIOSH Sound Level Meter app was previously known as the *NoiSee* app (version 1.0) in 2014. A study by Kardous and Shaw [6] showed that NoiSee app measurements were within 2 dB(A) of the noise levels of a sound source and concluded that the app was adequate for occupational noise assessment. Similarly, Crossley et al [20] evaluated the fit of 9 apps and concluded that the NIOSH Sound Level Meter app had the best fit, with an *R*^2^ value of 0.97. The SoundMeter X app was previously known as *SoundMeter* (version 3.3.1) in 2014. Nast et al [21] evaluated the accuracy of mobile apps and determined that the SoundMeter app had the highest accuracy, with a mean difference of −0.5 dB(A), and the narrowest variance distribution. Although the SoundMeter X app in our study was not the most accurate, its error could still be considered within the tolerable range. However, the app consistently underestimated actual sound levels, as reported in a study by McLennon et al [7], who also evaluated Decibel X (previously known as Decibel 10th [version 4.3.5]). In their study, the Decibel X app was used on an iPhone and showed high inconsistency in measuring sound levels ranging from 60 dB to 90 dB. A later study showed that the Decibel X app ranked at the bottom of a fit assessment, with an *R*^2^ value of 0.77 [20]. Therefore, the performance of the Decibel X app in our study is consistent with previous simulation studies.

### Limitations

There are several limitations and constraints to this study. In terms of hardware, only an iPhone 13 Pro, which was produced in 2022, was used for noise assessment via mobile apps. Sound measurements may differ between new hardware and old hardware. Further, SLM apps are limited by the microphone used [22]. Almost all smartphone devices are fitted with microelectromechanical system microphones, which, on a technical basis, have limitations in meeting the national and international requirements for sound measurement instrumentation. However, attaching a high-quality condenser microphone and preamplifier to professional SLMs allows them to conform to international standards, such as IEC 61672-1 [19]. Furthermore, in this study, the apps were not calibrated, and the experiments were designed to simulate actual use in an occupational noise mapping scenario.

In terms of app selection, this study only screened apps for iOS systems and did not search for apps for Android systems. A prior study compared the accuracy of SLM apps for both systems and concluded that iOS apps were more accurate [8]. In addition, there are many brands of Android phones, unlike Apple iPhones, which use fewer and uniform hardware (eg, microphones and chips), and this may influence study outcomes and translatability [7]. Nonetheless, for the purpose of completeness, research that systematically evaluates the accuracy and usefulness of SLM apps needs to extend to Android systems. In addition, only apps with data logging and exporting functions were selected for this study. In occupational settings however, there may only be a need to have a function for displaying measured values and not necessarily logging or exporting functions. Furthermore, the features of mobile apps may change with version updates. Therefore, the results of this study are only representative of the apps’ versions at the time of testing.

Noise levels in this study ranged from 45 dB to 85 dB for all measurement scenarios, but there was a lack of data for noise levels above 85 dB. Operator safety was considered in this study’s design. Furthermore, only 30-second LAeqs were evaluated throughout this study. Further studies could add other measurement metrics, such as time-weighted average values, for comparison and analysis.

The reliance on the subjective (expert) judgments of the evaluators limited the results of using the MARS in this research, and their judgments may not represent the views of workers or other potential users of the SLM apps. However, this issue was partially addressed by checking the internal reliability of the scores given by each independent evaluator. The inclusion of workers as users who lack expertise could easily influence decisions on product feature trade-offs. We compared the overall scores of the three apps, and the app that we considered the most accurate had the lowest MARS score, while the app that we considered the least accurate had the highest MARS score, although the relative differences between their overall scores were small. This suggests that the MARS may be more suited to assessing perceptions on the use of an app rather than an app’s usefulness. Furthermore, the MARS may be more suited to information-based apps that collect, record, and store information, such as guides for chemical hazard management or ergonomic assessment tools.

### Conclusions

This study examined and assessed the accuracy and usefulness of SLM mobile apps. Under the conditions of this study, the NIOSH Sound Level Meter app had equivalent accuracy to the calibrated conventional SLM and demonstrated a degree of usefulness according to relevant expert judgments. This suggests that the NIOSH Sound Level Meter app may be suitable for monitoring noise levels in scenarios where cost prohibits the purchase or use of a conventional SLM device or where the rapid evaluation of noise-reducing control measures is required to reduce the risk of exposure. Moreover, the mobile app should complement but not replace conventional SLMs, especially when trying to determine worker risk. Lastly, the MARS may have limited applicability to measurement-based apps and may be more suited to information-based apps that collect, record, and store information. Future research assessing the usefulness of other occupational health and safety apps involving measurement (eg, light and heat measurement) should consider MARS outcomes in conjunction with accuracy measurements when determining the suitability of apps for occupational health and safety management.

## Supplementary material

10.2196/46846 Multimedia Appendix 1 Specifications of the three sound level meter apps included in this study.

10.2196/46846 Multimedia Appendix 2 Detailed scores from the three health specialists (deidentified by participant number) for the overall usefulness of the three mobile sound level meter apps, namely SoundMeter X, Decibel X, and NIOSH (National Institute for Occupational Safety and Health) Sound Level Meter.

## Abbreviations

dB(A): A-weighted dB
IEC: International Electrotechnical Commission
LAeq: A-weighted equivalent continuous sound level
MARS: Mobile App Rating Scale
NIOSH: National Institute for Occupational Safety and Health
ONIHL: occupational noise-induced hearing loss
SLM: sound level meter
