# Supplementary material for: Assessing the Usefulness of Mobile Apps for Noise Management in Occupational Health and Safety: Quantitative Measurement and Expert Elicitation Study
Source: JMIR Mhealth Uhealth. 2023 Nov 14;11:e46846. doi: 10.2196/46846 (PMC10686533; doi:10.2196/46846)
Supplement: Multimedia Appendix 1 [file mhealth-v11-e46846-s001.docx]

## Multimedia Appendix 1

| App | Version | App Store Rating | Developer | Logging Ability |
| --- | --- | --- | --- | --- |
| **NIOSH** | (iOS) 1.2.5 | 4.6 | The National Institute for Occupational Safety and Health (NIOSH) | Yes |
| **Decibel X** | (iOS) 9.5.0 | 4.5 | SkyPaw Co. Ltd | Yes |
| **SoundMeter X** | (iOS) 11.5.6 | 2.5 | Faber Acoustical, LLC | Yes |
