# Supplementary material for: Assessing the Usefulness of Mobile Apps for Noise Management in Occupational Health and Safety: Quantitative Measurement and Expert Elicitation Study
Source: JMIR Mhealth Uhealth. 2023 Nov 14;11:e46846. doi: 10.2196/46846 (PMC10686533; doi:10.2196/46846)
Supplement: Multimedia Appendix 2 [file mhealth-v11-e46846-s002.docx]

## Multimedia Appendix 2

| **Participant number** | | **1742** | | | **1632** | | | **1386** | | |
| --- | --- | --- | --- | --- | --- | --- | --- | --- | --- | --- |
| **Mobile app** | | **SoundMeter X** | **Decibel X** | **NIOSH** | **SoundMeter X** | **Decibel X** | **NIOSH** | **SoundMeter X** | **Decibel X** | **NIOSH** |
| Engagement | Entertainment | 3 | 3 | 2 | 4 | 5 | 2 | 3 | 5 | 3 |
|  | Interest | 4 | 4 | 2 | 5 | 5 | 3 | 3 | 5 | 4 |
|  | Customisation | 4 | 4 | 4 | 5 | 5 | 3 | 3 | 4 | 5 |
|  | Interactivity | 3 | 4 | 3 | 4 | 4 | 2 | 3 | 4 | 4 |
|  | Target group | 3 | 4 | 3 | 5 | 5 | 4 | 4 | 5 | 5 |
| Functionality | Performance | 4 | 3 | 3 | 5 | 4 | 4 | 4 | 5 | 5 |
|  | Ease of use | 3 | 3 | 3 | 3 | 4 | 5 | 4 | 4 | 5 |
|  | Navigation | 3 | 2 | 4 | 4 | 5 | 5 | 4 | 4 | 5 |
|  | Gestural design | 4 | 3 | 4 | 3 | 5 | 4 | 4 | 5 | 5 |
| Aesthetic | Layout | 4 | 2 | 3 | 3 | 4 | 4 | 4 | 5 | 5 |
|  | Graphics | 4 | 3 | 2 | 3 | 5 | 3 | 3 | 5 | 4 |
|  | Visual appeal | 4 | 4 | 1 | 4 | 5 | 3 | 3 | 5 | 4 |
| Information quality | Accuracy | 4 | 3 | 3 | 3 | 4 | 5 | 5 | 5 | 5 |
|  | Goals | 4 | 3 | 3 | 4 | 4 | 0 | 4 | 4 | 5 |
|  | Quality of information | 4 | 1 | 5 | 3 | 5 | 5 | 4 | 5 | 4 |
|  | Quantity of information | 2 | 2 | 5 | 4 | 4 | 5 | 3 | 4 | 5 |
|  | Visual info | 3 | 1 | 4 | 4 | 4 | 4 | 4 | 5 | 4 |
|  | Credibility | 5 | 3 | 5 | 4 | 4 | 5 | 3 | 4 | 4 |
| Subjective quality | Recommendation | 4 | 3 | 4 | 4 | 5 | 4 | 3 | 5 | 4 |
|  | How many times use | 3 | 2 | 3 | 4 | 4 | 3 | 3 | 4 | 4 |
|  | Would you pay | 5 | 1 | 1 | 1 | 5 | 3 | 1 | 5 | 3 |
|  | Overall rating | 4 | 4 | 4 | 4 | 5 | 3 | 3 | 5 | 4 |
